# A pipeline for sample tagging of whole genome bisulfite sequencing data using genotypes of whole genome sequencing

**DOI:** 10.1186/s12864-023-09413-2

**Published:** 2023-06-23

**Authors:** Zhe Xu, Si Cheng, Xin Qiu, Xiaoqi Wang, Qiuwen Hu, Yanfeng Shi, Yang Liu, Jinxi Lin, Jichao Tian, Yongfei Peng, Yong Jiang, Yadong Yang, Jianwei Ye, Yilong Wang, Xia Meng, Zixiao Li, Hao Li, Yongjun Wang

**Affiliations:** 1 Department of Neurology, Beijing Tiantan Hospital, Capital Medical University, Beijing, 100070 China; 2 China National Clinical Research Center for Neurological Diseases, Beijing, 100070 China; 3 Center of excellence for Omics Research (CORe), Beijing Tiantan Hospital, Capital Medical University, Beijing, 100070 China; 4 Clinical Center for Precision Medicine in Stroke, Capital Medical University, Beijing, 100069 China; 5 Advanced Innovation Center for Human Brain Protection, Capital Medical University, Beijing, 100069 China; 6 BioChain (Beijing) Science and Technology, Inc, Economic and Technological Development Area, 100176 Beijing, P. R. China

**Keywords:** Sample tagging, Whole genome bisulfite sequencing, Whole genome sequencing, Genetic variants, Multi-omics

## Abstract

**Background:**

In large-scale high-throughput sequencing projects and biobank construction, sample tagging is essential to prevent sample mix-ups. Despite the availability of fingerprint panels for DNA data, little research has been conducted on sample tagging of whole genome bisulfite sequencing (WGBS) data. This study aims to construct a pipeline and identify applicable fingerprint panels to address this problem.

**Results:**

Using autosome-wide A/T polymorphic single nucleotide variants (SNVs) obtained from whole genome sequencing (WGS) and WGBS of individuals from the Third China National Stroke Registry, we designed a fingerprint panel and constructed an optimized pipeline for tagging WGBS data. This pipeline used Bis-SNP to call genotypes from the WGBS data, and optimized genotype comparison by eliminating wildtype homozygous and missing genotypes, and retaining variants with identical genomic coordinates and reference/alternative alleles. WGS-based and WGBS-based genotypes called from identical or different samples were extensively compared using hap.py. In the first batch of 94 samples, the genotype consistency rates were between 71.01%-84.23% and 51.43%-60.50% for the matched and mismatched WGS and WGBS data using the autosome-wide A/T polymorphic SNV panel. This capability to tag WGBS data was validated among the second batch of 240 samples, with genotype consistency rates ranging from 70.61%-84.65% to 49.58%-61.42% for the matched and mismatched data, respectively. We also determined that the number of genetic variants required to correctly tag WGBS data was on the order of thousands through testing six fingerprint panels with different orders for the number of variants. Additionally, we affirmed this result with two self-designed panels of 1351 and 1278 SNVs, respectively. Furthermore, this study confirmed that using the number of genetic variants with identical coordinates and ref/alt alleles, or identical genotypes could not correctly tag WGBS data.

**Conclusion:**

This study proposed an optimized pipeline, applicable fingerprint panels, and a lower boundary for the number of fingerprint genetic variants needed for correct sample tagging of WGBS data, which are valuable for tagging WGBS data and integrating multi-omics data for biobanks.

**Supplementary Information:**

The online version contains supplementary material available at 10.1186/s12864-023-09413-2.

## Background

Advances in sequencing technologies have greatly reduced the costs of massively parallel sequencing, enabling large-scale whole genome sequencing (WGS) studies of healthy people and patients for investigating population structures, evolutionary adaptations, and genetic architectures of complex diseases such as ischaemic cerebrovascular disease [1, 2]. While genomic data analysis has identified several susceptible and disease-causing genes [3-7], the integration of multi-omics data offers a better understanding of the molecular pathophysiology and the discovery of new therapeutic targets or biomarkers for ischaemic cerebrovascular disease [8, 9]. In this era of large-scale sequencing, conducting multi-omics analyses for tens of thousands of individuals would become standard practice, making sample tagging a vital quality control procedure. Accurate sample tagging prevents sample mix-ups, reduces false positives/negatives, and increases the reproducibility of subsequent bioinformatics analyses [10, 11]. This involves tagging each sample with a unique combination of fingerprint variant genotypes. Currently, many panels of fingerprint variants have been proposed for sample tagging of DNA genomic data by comparing genotypes of the fingerprint variants generated using WGS and other methods [12-14]. In contrast, very few panels have been proposed to check sample identities of multi-omics data, such as epigenomics data. Until now, only 1 panel of 50 fingerprint SNPs has been published for sample tagging of the transcriptomic data [15]. Personal Genome Project-UK (PGP-UK) applied the 65 control SNPs on the Illumina HumanMethylation450 BeadChip array to tag whole genome bisulfite sequencing (WGBS) data, DNA methylation array data, and WGS data [16]. However, the PGP-UK study neither showed detailed protocols for WGBS sample tagging nor systematically evaluated the performance of the 65-SNP panel, which only provided limited guidance for integrating WBGS and WGS data. Currently, there is no established pipeline or optimized panel available for tagging WGBS data with the aid of WGS data. This is a critical need for multi-omics data integration and biobank constructions in large-scale sequencing projects.

Taking advantage of WGS and WGBS for identical patients of ischaemic cerebrovascular disease in the Third China National Stroke Registry (CNSR-III) [17], we solved the problem of correctly integrating WGBS and WGS data by designing a fingerprint panel of autosome-wide A/T polymorphic single nucleotide variants (SNVs) and constructing an optimized pipeline for sample tagging of WGBS data. WGS-based and WGBS-based genotypes called from identical or different samples were extensively compared within a first batch of 94 samples and then within a second batch of 240 samples. Moreover, to figure out the lower limit for the number of fingerprint variants in the panel that was capable to tag WGBS data using this pipeline, we also explored the performance of another 6 fingerprint panels, and the lower limit was further validated using 2 self-designed fingerprint panels. Taken together, this study systematically investigated sample tagging of WGBS data using genotypes of WGS, and provided a pipeline and a few applicable fingerprint panels. Their application would help to integrate WGBS and WGS data of large-scale sequencing projects.

## Results

### Identification of 94 samples with correct identities

To construct a pipeline for sample tagging of WGBS data, we randomly selected 94 samples of the CNSR-III cohort that underwent WGS and WGBS. To ensure that the DNA samples were not mistaken during WGS and WGBS, genotyping of 52 biallelic fingerprint SNPs using mass spectrometry was independently carried out before WGBS (Methods), and genotypes of the 52 SNPs were compared between WGS and mass spectrometry data for each sample. Because sample identities have been strictly checked and stringent quality control was applied during the WGS project [18], genotypes extracted from WGS data were used as truth data here. Using normal procedures of genotype comparison by hap.py software, it was shown that for each of the 94 samples, either precision or recall was ≥ 0.95 (Supplementary Table 2). The lowest number of true positives (TP) genotypes was 22, mainly due to the low call rate for Sample 38 in the mass spectrometry experiment. While the number of TP genotypes for the other 93 samples was ≥ 30. Regarding the theoretical potentiality to discriminate 4.2 million (≈ 2^22^, because hap.py did not apply variants with 0/0 or./. genotypes in its calculation) for the TP genotypes and the differences in genotyping technologies between WGS and mass spectrometry, it would be safe to consider that the 94 samples were not mistaken in DNA sample transport, WGBS, mass spectrometry, and data delivery. And these 94 samples would be applied to test the pipeline for sample tagging of WGBS data using genotypes of WGS.

### Constructing an optimized pipeline for sample tagging of WGBS data using autosome-wide A/T polymorphic SNVs

**Fig. 1:**
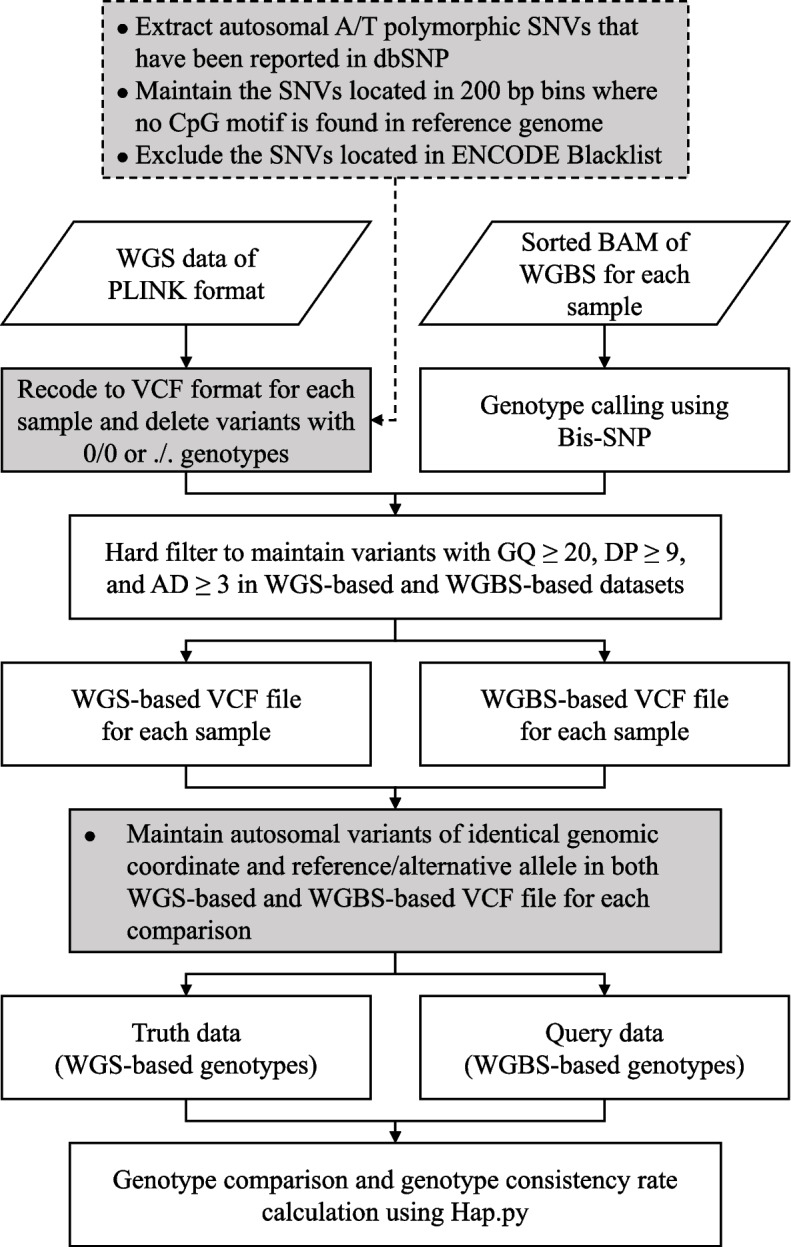
Pipeline for sample tagging of WGBS data. Gray-filled boxes represented optimizations for sample tagging of WGBS data. Specifically, gray-filled boxes with dotted borders showed the autosomal A/T polymorphic SNVs selection process for Fingerprint Panel 7. It should be noted that this process was not applicable for Fingerprint Panels 1-6 and 8-9 when using this pipeline

To tag samples with WGBS data, we constructed a pipeline to compare WGS-based and WGBS-based genotypes for each individual (Fig. 1). Bis-SNP was applied in the genotype calling of WGBS data [19]. Because Bis-SNP did not output genetic variants with wildtype homozygous genotype (0/0) and missing genotype (./.) in the WGBS-based VCF file, and hap.py did not utilize such genotypes in its calculation, we also eliminated genetic variants with these genotypes in WGS-based VCF file that was obtained after joint calling. Then variants with identical genomic coordinates and ref/alt alleles were extracted from WGS- and WGBS-based genotype data. Thus, an identical set of genetic variants was contained in the truth and query VCF files for genotype comparison.

Next, we designed a panel for this pipeline. Because no prior knowledge was available about to what extent the non-specific or incomplete conversion of unmethylated cytosines (C) to uracil (U) during bisulfite treatment would influence the accuracy of variant genotype calling of Bis-SNP, we took full advantage of the available WGS data and established a fingerprint panel consisted of 1,309,760 autosomal A/T polymorphic SNVs (Fingerprint Panel 7 in Table 1).

**Table 1:** Fingerprint panels that were investigated in this study

Fingerprint panel index	Origin/reference	Application of the panel	Number of variants	Number of autosomal variants	Number of autosomal variants captured by WGS of CNSR-III
1	Self-designed	DNA sample identification	52	52	52
2	Illumina HumanMethylation450 BeadChip array [20]	Sample identification for DNA methylation array	65	56	54
3	[21]	DNA sample identification	169	136	126
4	[13]	DNA sample identification and kinship analysis	448	336	321
5	[12]	DNA sample identification	1245	1218	1093
6	Affymetrix Genome-Wide Human SNP Array 6.0 [22]	Genotyping and chromosomal aberration analysis	929,867	890,404	756,584
7	Self-designed autosome-wide A/T polymorphic SNVs	Sample identification for WGBS	67107200^a^	63881625^a^	1309760^b^
8	Self-designed autosomal A/T polymorphic common SNVs	Sample identification for WGBS	1351	1351	1351
9	Self-designed autosomal common SNVs	Sample identification for WGBS	1278	1278	1278

^a^ calculated using genetic variants data of dbSNP

^b^ filtered by the 200 bp bin where no CpG motif was found and ENCODE Blacklist of the human genome (see Methods)

**Fig. 2:**
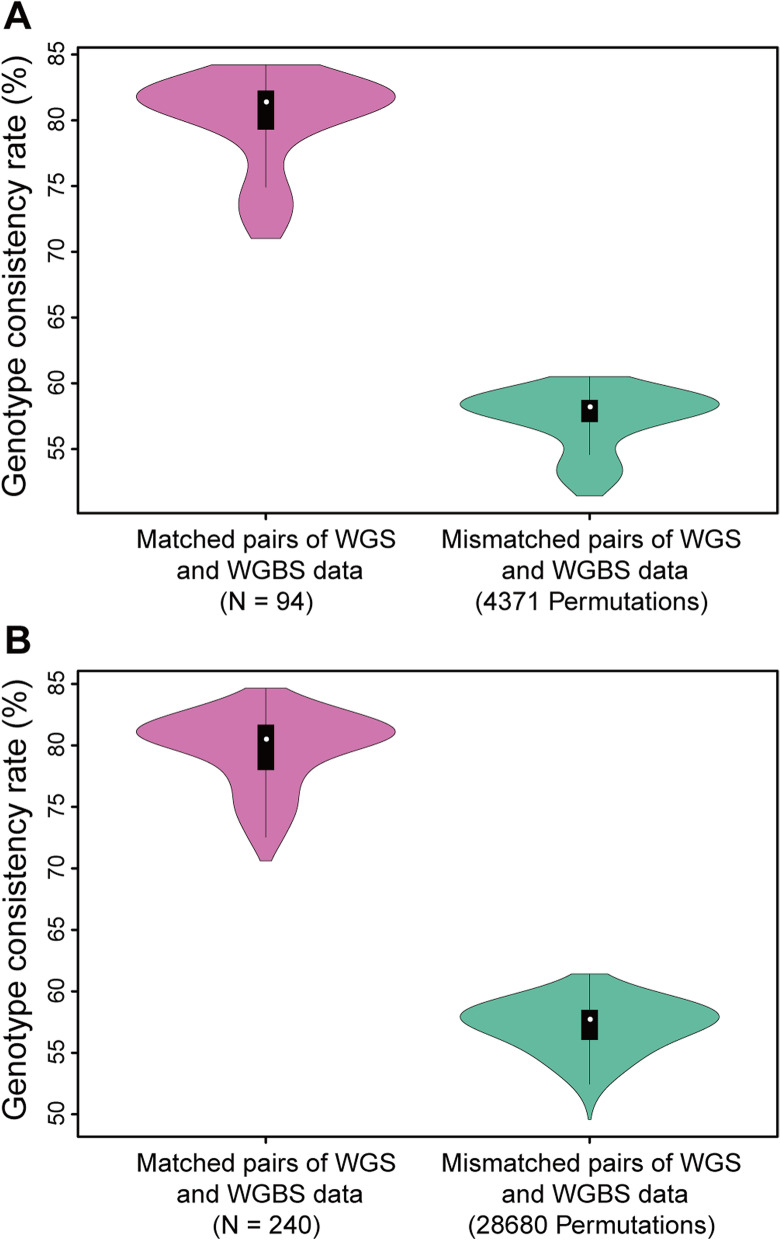
Violin plots for genotype consistency rate of Fingerprint Panel 7. **A** Genotype consistency rate among the 94 samples in the first batch. **B** Genotype consistency rate among the 240 samples in the second batch. Genotype consistency rate of matched pairs of WGS and WGBS data was shown in pink, while genotype consistency rate of mismatched pairs of WGS and WGBS data (exhaustive permutation) was shown in light green

For the first batch of 94 samples, the genotype consistency rates for the 94 correctly matched pairs of WGS and WGBS VCF files were above 70% (ranging from 71.01% to 84.23%, Fig. 2A, Table 2). In contrast, the genotype consistency rate for mismatched pairs of WGS and WGBS data was all below 70% (ranging from 51.43% to 60.50%, Fig. 2A, Table 2) after 4371 permutations. Therefore, a clear gap in genotype consistency rate naturally occurred and it could be applied to distinguish WGS-based and WGBS-based genotype calls of an identical sample from those of different samples.

**Table 2:** Median and range (in brackets) of genotype consistency rate between truth (WGS-based) and query (WGBS-based) VCF files for the 94 samples in the first batch

Fingerprint panel index	Genotype consistency rate (%)		*P*-value
	Matched pairs (*N* = 94)	Mismatched pairs (4371 permutations)	
1	91.83 [73.33–100.00]	58.82 [16.67–100.00]	< 2.2 × 10^–16^
2	82.76 [60.00–96.30]	57.14 [21.74–89.47]	< 2.2 × 10^–16^
3	85.61 [72.00–96.83]	58.18 [33.33–84.38]	< 2.2 × 10^–16^
4	86.39 [72.02–92.45]	59.26 [43.42–74.77]	< 2.2 × 10^–16^
5	86.15 [75.05–91.96]	59.87 [50.39–68.19]	< 2.2 × 10^–16^
6	81.61 [68.56–85.10]	57.92 [51.74–59.64]	< 2.2 × 10^–16^
7	81.39 [71.01–84.23]	58.20 [51.43–60.50]	< 2.2 × 10^–16^
8	85.57 [77.95–91.30]	60.80 [53.79–69.19]	< 2.2 × 10^–16^
9	86.39 [77.57–89.83]	61.70 [53.44–69.78]	< 2.2 × 10^–16^

The index of fingerprint panels was identical to that in Table 1. The genotype consistency rate ranges, displayed in the format of [minimum–maximum], were presented in brackets. *P*-value showed the significance of one-sided t-tests

### Validation of sample tagging for WGBS data among the second batch of 240 samples

To validate the capability of the pipeline and Fingerprint Panel 7 in sample tagging for WGBS data, we replicated the genotype comparisons within a second batch of 240 samples. Despite no prior genotyping of fingerprint SNPs for the 240 samples, executing the pipeline revealed that the genotype consistency rate between matched pairs of WGS and WGBS data was higher than 70% (ranging from 70.61% to 84.65%, Fig. 2B, Table 2), indicating that each pair of WGS and WGBS data came from the identical sample.

Then we exhaustively permutated the sample ID order and conducted 28,680 comparisons between mismatched pairs of WGS and WGBS data. As shown in Fig. 2B and Table 3, all of the genotype consistency rates were below 70% for the permutations (ranging from 49.58% to 61.42%). Therefore, the gap in genotype consistency rate between matched and mismatched pairs of WGS and WGBS data was validated, and the sample identities of WGBS data could be confirmed by executing the optimized pipeline using autosome-wide A/T polymorphic SNVs.

**Table 3:** Median and range (in brackets) of genotype consistency rate between truth (WGS-based) and query (WGBS-based) VCF files for the 240 samples in the second batch

Fingerprint panel index	Genotype consistency rate (%)		*P*-value
	Matched pairs (*N* = 240)	Mismatched pairs (28,680 permutations)	
1	91.55 [73.33–100.00]	59.09 [13.33–100.00]	< 2.2 × 10^–16^
2	82.14 [53.13–97.14]	56.00 [0.00–95.45]	< 2.2 × 10^–16^
3	85.07 [61.70–96.97]	58.00 [26.00–86.96]	< 2.2 × 10^–16^
4	85.71 [72.09–92.52]	59.29 [42.70–76.83]	< 2.2 × 10^–16^
5	86.32 [74.56–91.48]	60.30 [50.16–72.95]	< 2.2 × 10^–16^
6	81.42 [70.19–84.48]	57.59 [50.91–60.12]	< 2.2 × 10^–16^
7	80.51 [70.61–84.65]	57.72 [49.58–61.42]	< 2.2 × 10^–16^
8	85.09 [77.12–90.93]	60.87 [51.36–70.79]	< 2.2 × 10^–16^
9	86.54 [77.32–92.26]	61.80 [52.44–72.56]	< 2.2 × 10^–16^

The index of fingerprint panels was identical to that in Table 1. The genotype consistency rate ranges, displayed in the format of [minimum–maximum], were presented in brackets. *P*-value showed the significance of one-sided t-tests

### The lower limit of the order of magnitude for the number of genetic variants capable to tag WGBS data using the pipeline

In the above analyses, the sample identities of WGBS data were tagged by genotypes of autosome-wide A/T polymorphic SNVs, which were extracted from WGS data. For large-scale multi-omics studies, checking sample identities of WGBS data with autosome-wide A/T polymorphic SNVs is feasible because of the availability of both WGS and WGBS data. However, for epigenomics studies that only conduct WGBS, applying this method requires extra WGS, which could be unnecessary and expensive. Therefore, we searched for published fingerprint panels for DNA data sample tagging and evaluated their performance using the pipeline. The aim was to identify the lower limit of the order of magnitude for the number of genetic variants in the panel that is capable to tag sample identities of WGBS data.

Except for the autosome-wide A/T polymorphic SNVs, six panels (Fingerprint Panels 1–6) with the number of included genetic variants ranging from the order of tens, hundreds, and thousands to 900 K were tested using the pipeline (Table 1). Among the first batch of 94 samples, although the consistency rate between matched pairs of WGS and WGBS data was significantly higher (*P*-value < 2.2 × 10^–16^, one-sided t-test) than that of mismatched pairs for Fingerprint Panels 1–6 (Table 2), the distribution ranges of genotype consistency rate overlapped between matched and mismatched pairs of WGS and WGBS data for fingerprint panels with less than 1000 genetic variants (Fingerprint Panels 1–4). For these panels, no clear gap was evident to distinguish whether the genotype data were extracted from an identical sample or different samples (Table 2, Supplementary Fig. 1–4). In contrast, for Fingerprint Panels 5 and 6 with more than 1000 genetic variants, the gap in consistency rate between matched and mismatched pairs of WGS and WGBS data was demonstrated (Table 2, Supplementary Fig. 5–6). The performance of the Fingerprint Panels 1–6 was also tested among the second batch of 240 samples. The same phenomenon as in the first batch was reproduced, with the gap in genotype consistency rate only detected for panels with more than 1000 genetic variants (Table 3, Supplementary Fig. 1–6).

To validate this lower limit for genetic variants in fingerprint panels, we constructed 2 panels with slightly over 1000 common SNVs (Fingerprint Panels 8 and 9 in Table 1). As shown in Supplementary Fig. 7–8, the gap in genotype consistency rate was demonstrated for these 2 panels, indicating that fingerprint panels containing thousands of genetic variants can label sample identities for WGBS data using this pipeline.

In addition, we also tested the potential of the number of genetic variants in truth/query VCF files and the number of TP to tag WGBS data. Although it is widely accepted that the truth and query VCF files contain a greater number of genetic variants in genotype data extracted from WGS and WGBS samples of the same individual, none of the nine panels examined in this study provided clear evidence of the distribution of these numbers being separated between WGS and WGBS data of matched and mismatched pairs (Supplementary Tables 3–6, Supplementary Fig. 9–11). While a gap in the distribution of the number of TP among 94 samples from the first batch of Fingerprint Panel 5 was detected (Supplementary Table 4, Supplementary Fig. 9), the lack of validation for this gap in the second batch of samples (Supplementary Table 6) indicates that the number of TP is not useful in labeling WGBS data.

## Discussion

Sample tagging is an essential quality control procedure because it could help to eliminate the incorrect association between omics data and samples, reduce the risk of errors, and improves the accuracy and reproducibility of the results. Although WGBS was widely applied in medical and biological research, the methods for sample tagging of WGBS data have not been systematically investigated. Taking advantage of large-scale WGS and WGBS for stroke patients in CNSR-III, we constructed an optimized pipeline for sample tagging of WGBS data. A total of 9 panels, including one self-designed autosome-wide A/T polymorphic SNV panel, one genome-wide SNP genotyping array, five fingerprint panels for tagging DNA data, and two self-designed panels with the number of genetic variants slightly over 1000, were tested for the capability to tag WGBS data by executing the pipeline, and extensive permutations were conducted when comparing truth and query VCF files. The results showed that using the optimized pipeline, the genotype consistency rate for panels containing over 1000 genetic variants was able to distinguish WGS-based and WGBS-based genotype VCF files of an identical sample from those of different samples, and the capability of these panels to tag WGBS data was independently validated in 2 batches of samples.

Compared with sample tagging of DNA data, the sample tagging of WGBS data is particularly challenging due to the bisulfite conversion process. WGBS data bisulfite conversion occurs under acidic conditions and high temperatures, which could result in DNA degradation and the introduction of genotyping noise [23, 24]. Although bisulfite treatment is intended to convert unmethylated C to T in CpG islands, the incomplete or excessive conversion of methylated C in CpG islands, as well as the non-specific conversion of other nucleotides, would potentially reduce the accuracy of genotype calling with the WGBS data. Our study showed that the effect of bisulfite conversion on genotype calling was not only limited to CpG islands. For instance, for genome-wide SNP genotyping microarray (Fingerprint Panel 6), the lowest genotype consistency rate between matched pairs of WGS and WGBS VCF files was 68.56% (Tables 2 and 3). This finding suggested that bisulfite conversion influenced genotype calling across the entire genome. Moreover, for autosome-wide A/T polymorphic SNVs, the lowest genotype consistency rate between matched pairs of WGS and WGBS VCF files was 70.61%, suggesting that non-specific conversion by bisulfite treatment also affected genotype calling for A/T polymorphic SNVs. For the other panels, the genotype consistency rate between matched pairs of WGS and WGBS VCF files ranged from 70 to 95%. Although Bis-SNP was implemented in the pipeline to obtain accurate genotype calls from WGBS data [25], we suspect that genotype calling accuracy for genetic variants with all kinds of polymorphisms was uniformly affected by bisulfite treatment in WGBS. Furthermore, the impact of bisulfite treatment on genotype calling did not seem to be reduced by implementing the pipeline using autosome-wide A/T polymorphic SNVs, because the distribution of genotype consistency rate between matched pairs of WGS and WGBS VCF files for Fingerprint Panels 6 and 7 did not show significant differences (two-sided t-test, *P* value = 0.8941 and 0.1539, respectively for the first and second batch).

Tens or no more than one-hundred fingerprint genetic variants were sufficient to correctly tag genomic data from DNA genotyping or sequencing, which was conducted by simply counting the number of fingerprint genetic variants with identical genotypes between DNA profiles obtained from different methods or platforms [12, 13, 26]. In this scenario, three kinds of genotypes (0/0, 0/1, and 1/1) furnished useful information on sample identity. However, this “counting” method was inadequate for WGBS data tagging because bisulfite treatment affected genotype calling accuracy across all three kinds of genotypes, and the stability of experimental conditions of bisulfite treatment was not perfectly controlled for all WGBS samples. Therefore, the number of genetic variants with identical genotypes could be small between matched WGS and WGBS data if excessive bisulfite treatment was performed, and the number might be large between mismatched WGS and WGBS data in case of insufficient bisulfite treatment. The “counting method” may not be able to handle such complications in sample tagging. Although a few modifications were adopted by our pipeline compared with the traditional “counting method”, sample identities could not be verified by comparing the number of genetic variants with identical genotypes (Supplementary Tables 4 and 6, Supplementary Fig. 9–11). Therefore, we focused on calculating the genotype consistency rate rather than counting the number of TPs in WGBS data sample tagging in this study.

The reduced genotype calling accuracy when using WGBS data necessitated an increased number of fingerprint genetic variants to calculate the genotype consistency rate, and then to correctly tag the WGBS data. To benchmark a large number of genotype calls against the truth datasets, we employed hap.py software in our pipeline. Moreover, the number of genetic variants for the panels that were needed to correctly tag WGBS data was further increased because genetic variants with 0/0 or./. genotypes would be neither reported by Bis-SNP in WGBS-based VCF files nor utilized by hap.py. This study showed that at least more than 1000 fingerprint variants were required to correctly tag WGBS data, in contrast, for fingerprint panels with less than 1000 genetic variants, the genotype consistency rate was not separated between matched and mismatched pairs of WGS-based and WGBS-based VCF files. For Fingerprint Panel 1, the highest genotype consistency rate of mismatched pairs of WGS-based and WGBS-based VCF files was 100% (Tables 2 and 3), which was observed for 3 pairs of mismatched WGS-based and WGBS-based VCF files. It was found that the 3 comparisons only utilized 8, 10, and 11 SNPs. When the SNPs in the fingerprint panel are common SNPs with high minor allele frequency (MAF), there is a high probability that 2 unrelated individuals carry identical genotypes at these 8–11 loci. The genotype comparisons of the 3 mismatched pairs could be regarded as a counterexample for WGBS data tagging, and increasing the number of fingerprint variants in the panel could help to correctly tag WGBS data.

In this study, Fingerprint Panel 2, a 65-SNP panel in Illumina HumanMethylation450 BeadChip array, had been applied to check the sample identities in PGP-UK [16]. The mean agreement between genotypes of WGS and WGBS was 99.45% in that study for a pilot cohort of 10 samples. Although their genotype consistency rate is significantly higher than that of this study, several factors might account for this difference. Firstly, the use of different library construction kits, polymerases, and bisulfite conversion protocols in the two studies may have led to varying degrees of DNA damage, affecting the accuracy of variant calling [27]. Secondly, PGP-UK used the gemBS software [28], which adopted differing variant calling algorithms compared to Bis-SNP used in our study. Thirdly, we also evaluated gemBS and found it superior to Bis-SNP in that, for variants with C or G as ref alleles, it can call wild-type homozygous genotypes. This is important in “counting method”-based sample tagging, and suggests that PGP-UK might have utilized a different pipeline than our study did. Lastly, by not shuffling the sample order to perform exhaustive permutation during the evaluation of the 65-SNP fingerprint panel, it is challenging to confirm that the pilot cohort’s ten samples were correctly tagged for WGBS and WGS data. Hence, we contend that PGP-UK’s and our study’s genotype consistency rates were non-comparable unless PGP-UK provides more details on library construction, sample tagging methods and pipelines, and the performance evaluation of the 65-SNP fingerprint panel on mismatched sample pairs. Moreover, it was demonstrated in our study that this panel could not tag WGBS data using our pipeline.

The genotype consistency rates for matched WGS and WGBS data were over 70% for fingerprint panels with over 1000 genetic variants in this study. We conducted thorough literature searches using the Web of Science (Core collection) and PubMed databases to find the level of genotype consistency in the current field. Unfortunately, limited attention has been given to addressing the problem of sample tagging in WGBS data either due to the low sample sizes of previous WGBS studies or the less pressing need for WGBS data integration with WGS data. We did not find any studies, besides ours, that carried out a comparative analysis of WGBS data sample tagging. In literature searches on PubMed, three similar articles of PGP-UK reported methods for integrating multi-omics data [16]. However, after meticulous reading, one of the three articles proposed a robust approach to multi-omics data matching using epigenomic data from the Illumina HumanMethylation450 BeadChip from TCGA, rather than the WGBS technique [29]. The other two articles did not incorporate epigenomic or WGBS data or address the WGBS data sample tagging problem [30, 31].

For multi-omics data integration other than WGBS data, one study used a fingerprint panel of 50 SNPs to tag transcriptomic (RNASeq) data [15]. Despite applying the “counting method” in RNASeq data sample tagging, the study did not provide a genotype consistency rate. However, based on the number of TP in Fig. 6 of that study [15], the genotype consistency rate was calculated to be ranging from 75 to 100%.

This study has a few limitations. Firstly, although exhaustive comparisons were conducted to evaluate the capability to tag WGBS data for the fingerprint panels, the sample size was small compared to biobanks. Additionally, the performance of the panels should be further validated in tens of thousands of individuals. Secondly, the universality of the pipeline and fingerprint panels was not evaluated in this study. Different procedures in library construction and bisulfite conversion would introduce bias to WGBS, as mentioned earlier [27]. The influence on genotype calling using WGBS data and sample tagging was not investigated either. Although a gap in genotype consistency rate between matched and mismatched WGS and WGBS data was demonstrated, we were not sure whether the pipeline was compatible with other library construction methods of WGBS, such as that was used in PGP-UK. Therefore, no threshold or cutoff was proposed for sample tagging of WGBS data in this study. Thirdly, as shown in this study, fingerprint panels with at least 1000 genetic variants could be capable to tag WGBS data. In practice, the application of these panels also demanded extra targeted capture and high-throughput sequencing [12]. Further optimizations of the pipeline and fingerprint panels with enhanced capability were necessary. Fourthly, the steps in the pipeline were slightly complicated and would benefit from further optimization and simplification. For example, an identical set of genetic variants was applied in genotype comparison in this pipeline, which added a variant extraction operation before applying hap.py. Removing this optimization would only cause minor differences between precision and recall and would not significantly change the tagging results for the two batches of samples included in this study. However, under extreme conditions, this difference would be significant, hence identifying the reason would decrease the efficiency of large-scale WGBS data tagging. Regardless of these limitations, this study proposed a method that can successfully tag WGBS data, and the application of the pipeline could facilitate multi-omics data integration and biobank construction for common diseases, such as stroke [9], in the current omics era.

## Conclusions

We proposed an optimized pipeline for WGBS data sample tagging, and after rigorous comparisons, identified some applicable fingerprint panels. A lower limit on the number of genetic variants required to correctly tag WGBS data was identified to be in the thousands. The pipeline and panels presented in this study could assist in the future design and optimization of fingerprint panels for tagging WGBS data and benefit multi-omics data integration in biobanks.

## Methods

### Sample collection and WGS

DNA samples were obtained from the CNSR-III [17], a nationwide prospective registry for patients presented to hospitals with acute ischaemic cerebrovascular events between August 2015 and March 2018 in China. Written informed consent was obtained from all patients or legally authorized representatives before entering the study. WGS was conducted during 2019–2020 at BGI Genomics (BGI-Shenzhen) [18]. The WGS data were then processed under the Genome Analysis Toolkit (GATK) best practice guidance using Sentieon [32]. All of the reads were mapped to the non-N reference sequence of genome build GRCh38. Base Quality Score Recalibration (BQSR) was performed for each GVCF file, and Variant Quality Score Recalibration (VQSR) was conducted for quality control after joint genotype calling. Multiallelic variants were eliminated, and for each variant, the genotype for an individual was qualified if the depth (DP) was ≥ 9, and genotype quality (GQ) was ≥ 20. For heterozygous variants, allele depth (AD) should be ≥ 3. Otherwise, the genotype was set to missing. In this study, genetic variants on sex chromosomes were not used. After further examinations on DNA contamination, sample identity, and kinship relationship inference, WGS data of 10,241 unrelated samples were obtained (under review). Among them, two batches of randomly selected samples (N = 94 and 240 for the first and second batches, respectively) were applied to evaluate the performance of different sample tagging panels on WGBS data.

### Genotyping using mass spectrometry technology for a fingerprint panel consisting of 52 biallelic SNPs

To make sure that the DNA samples would not be mistaken during WGBS of the first batch, we selected 52 biallelic fingerprint SNPs (Fingerprint Panel 1 in Table 1). These 52 SNPs distribute on 18 different autosomes and are on average 17.41 Mb apart. The MAFs of these SNPs range from 0.33–0.5 within the Chinese samples in the 1000 Genome Project Phase 3 (1KGP3) high-depth dataset (*N* = 301, Supplementary Table 1) [33]. The variant genotype in the 1KGP3 high-depth dataset was subjected to the identical hard filter of DP, GQ, and AD as the CNSR-III WGS genotypes.

All of the 94 samples in the first batch were genotyped at these 52 SNPs. For each sample, approximately 30 ng of qualified genomic DNA is used. Locus-specific PCR and detection primers are designed using the MassARRAY Assay Design software (Agena Bioscience, CA, USA). Multiplex PCR and locus-specific single-nucleotide extension were performed for each DNA sample, then the products are desalted and transferred to a 384-well SpectroCHIP array. After MALDI-TOF (matrix-assisted laser desorption/ionization-time of flight) mass spectrometry, MassArray Typer software (v4.1, Agena Bioscience, CA, USA) was used to call the genotype for each participant.

### Genotype comparisons between truth and query genotypes

In this study, hap.py (https://github.com/Illumina/hap.py) was applied to check sample identities by calculating the precision and recall between truth and query genotypes in VCF format.

By default, true-positives (TP), false-positives (FP), false-negatives (FN), recall, and precision were defined as follows:

- TP: variants/genotypes that match in truth and query calls;
- FP: variants that have mismatching genotypes or alt alleles, as well as query variant calls in regions a truth set would call confident hom-ref regions;
- FN: variants present in the truth set, but missed in the query.
- Recall = TP/(TP + FN).
- Precision = TP/(TP + FP).

For the 94 samples that underwent Fingerprint Panel 1 genotyping with mass spectrometry, we compared the WGS-based genotype (truth) to the mass spectrometry-based genotype (query) using normal (unoptimized) procedures of hap.py to check sample identities.

To check sample identities of WGBS, genotypes that were called from WGBS data were applied in query VCF, while genotypes that were extracted from WGS data or called from mass spectrometry were applied in truth VCF in this study.

### Whole genome bisulfite sequencing (WGBS)

WGBS of samples in the CNSR-III began in the middle of 2021. Genomic DNA was extracted from the unrelated samples of CNSR-III using magnetic bead method on AE2130-96 automated nucleic acid exaction system (HollyCon Medical Technology Co., Ltd., Beijing, China). The concentration of genomic DNA was quantified using Qubit 3.0 fluorometer and NanoDrop 2000 (Thermo Scientific Co, Massachusetts, USA). Electrophoresis was conducted on 1% agarose gel to make sure that the majority of genomic DNA segments were longer than 20 Kb and were not substantially degraded. Genomic DNA samples with a concentration ≥ 12.5 ng/μL and a total amount ≥ 0.5 μg were qualified for further procedures.

The qualified genomic DNA (0.5 μg) and control non-methylated λ-phage DNA (Promega, Wisconsin, USA) was randomly fragmented by ultrasound using Covaris LE220 (Covaris, Massachusetts, USA) according to the manufacturer’s instructions. The DNA fragment peak was about 350 bp. The fragmented DNA was selected by Agencourt AMPure XP beads (Beckman Coulter, Florida, USA). The end-repair for DNA fragments was performed by adding an ‘A’ nucleotide to the 3’ end of each strand. Afterward, the dTTP-tailed methylated adapters were ligated to both ends of the repaired/dA-tailed DNA fragments. The ligation product was purified by DNA Clean & Concentrator-5 Kit (Zymo Research, California, USA). Then the purified product was subjected to bisulfite conversion using EZ DNA Methylation-Lightning Kit (Zymo Research, California, USA). Afterward, the products were amplified by PCR and then purified by Agencourt AMPure XP beads (Beckman Coulter, Florida, USA). The purified PCR products with a total mass ≥ 200 ng, and the main peak in 300 to 700 bp would be applied. The resulting libraries were pooled and sequenced on Illumina NovaSeq 6000 sequencer with paired-end 150 bp reads (2 × 150 bp), generating at least 90 Gb data per sample. The average depth for each subject was intended to be greater than 30×.

### Quality control and read alignment of WGBS data

FastQC (https://www.bioinformatics.babraham.ac.uk/projects/fastqc/) was used to evaluate the quality of WGBS reads according to Phred quality score, GC content, adapter content, and overrepresentation analysis. Adapter sequences were trimmed using FASTP with a minimum length of 36 bases and forced poly-G trimming [34]. Reads that met the following criteria were kept for further analysis: 1) more than 50% of bases had Phred quality score ≥ 19; 2) the number of N bases ≤ 5.

Adapter-trimmed reads were aligned to the human reference genome (build GRCh38) using BISMARK v0.23.0 and bowtie2 [35, 36]. BAM files were position-sorted using samtools and deduplicated using deduplicate_bismark with default parameters [36, 37].

### WGBS-based genotype calling

The genotype calling using whole-genome bisulfite sequencing data was conducted using Bis-SNP [19], a package employing the Genome Analysis Toolkit (GATK) map-reduce framework. Bis-SNP is known for its precision in genotyping using bisulfite-treated massively parallel sequencing with Illumina directional library protocol [25]. The calling was performed under the default parameter setting of Bis-SNP guidelines (https://people.csail.mit.edu/dnaase/bissnp2011/BisSNP-UserGuide-latest.pdf). The genotype of a genetic variant was regarded to be qualified if the DP was ≥ 9, and further for heterozygous variants, the AD should be ≥ 3.

### A panel of autosome-wide A/T polymorphic SNVs

To reduce the influence of non-specific and incomplete conversion of unmethylated cytosines (C) to uracil (U) in bisulfite treatment on sample tagging of WGBS data, we constructed a fingerprint panel for tagging WGBS data using all of the A/T polymorphic SNVs in autosomes that fulfilled the following criteria (Fingerprint Panel 7 in Table 1): 1) the reference and alternative allele of the SNV must A and T in WGS, WGBS, and dbSNP (https://ftp.ncbi.nih.gov/snp/.redesign/.archive/b155/VCF/GCF_000001405.39.gz); 2) the human reference genome was divided into consecutive bins of 200 bp in length, and qualified A/T polymorphic SNVs should be located in bins that no CpG motif was found in its 200 bp bin; 3) A/T polymorphic SNVs in ENCODE Blacklist of the human genome were omitted [38]. All of the qualified autosomal A/T polymorphic SNVs were included in Fingerprint Panel 7 of this study.

### Construction of 2 fingerprint panels with about 1000 common SNVs

To validate the lower limit for the number of genetic variants that were required to tag WGBS data using the pipeline, we constructed Fingerprint Panels 8 and 9 (Table 1). Both panels contained slightly more than 1000 common SNVs.

Fingerprint Panel 8 consists of A/T polymorphic SNVs with MAFs ≥ 0.35, call rate ≥ 0.95, and *P*-value for Hardy–Weinberg Equilibrium > 10^–6^ in both 10,241 unrelated samples of CNSR-III and 301 Chinese samples in 1KGP3 high-depth dataset. Then, A/T polymorphic SNVs located in bins that had CpG motif in its 200 bp bin and ENCODE Blacklist of the human genome were excluded. We performed linkage disequilibrium (LD) pruning separately for each population (10,241 unrelated samples from the CNSR-III and 301 Chinese samples in 1KGP3) using an R^2^ < 0.01 in a sliding window of 500 Kb with a 1 SNV step. The Fingerprint Panel 8 was composed of the overlapping SNVs in both populations.

We used a similar approach to create Fingerprint Panel 9, but we did not restrict the selection of SNVs to A/T polymorphic SNVs, and we did not exclude SNVs in the CpG-motif-containing 200 bp bin or ENCODE Blacklist. After LD pruning, the intersection of the remaining SNVs for the two populations yielded Fingerprint Panel 9.

### Construction of a pipeline for sample tagging of WGBS data

In this study, the sample tagging of WGBS data is accomplished by comparing WGS-based genotypes and WGBS-based genotypes for each individual using hap.py. As shown in Fig. 1, compared with normal sample tagging of DNA sequencing or genotyping data, optimization for sample tagging of WGBS data included: 1) genetic variants with wildtype homozygous genotype (0/0) and missing genotype (./.) would be deleted before comparison; 2) for each pair of truth and query VCF files, genetic variants with identical genomic coordinates and reference/alternative alleles (intersection of genetic variants for the 2 VCF files) would be reserved for genotype comparison. After these operations, an identical set of genetic variants would be applied in the comparison between truth and query genotype VCF files. Notably, the truth and query VCF files had no genetic variants with 0/0 or./. genotypes, resulting in numerical equality of recall and precision. Consequently, this numerical equality was denoted as the genotype consistency rate in this study and was utilized to verify sample identities.

### Evaluation of different sample tagging panels

In this study, a total of 9 fingerprint panels (Table 1) were applied to check the sample identities of WGBS data. The Fingerprint Panels 2–6 were obtained via literature search, and the number of genetic variants in these 5 panels ranged from 65 to more than 900 K. Fingerprint Panels 1–5 were only proposed to verify sample identities in DNA sequencing and genotyping data. Fingerprint Panel 6 was Affymetrix Genome-Wide Human SNP Array 6.0, and was applied in genome-wide SNP genotyping.

For all of the 9 panels, their capability for sample tagging of WGBS data was tested among the first batch of 94 samples and then validated in the second batch of 240 samples.

### Permutation of samples

To obtain and validate the potential thresholds for genotype consistency rate in WGBS data tagging, we not only compared truth and query genotypes that were obtained from data with identical sample ID (denoted as matched pairs in this study), but also compared all pairs of truth and query genotypes that were respectively obtained from WGS and WGBS data with different sample IDs (denoted as mismatched pairs) by permutation. Exhaustive permutation of sample IDs was carried out respectively for the 2 batches. For each batch, the samples’ names were sorted alpha-numerically, and the WGS-based genotype of the first sample in the sorted list was used as the truth VCF file, and the WGBS data of the remaining samples in the batch were used as query VCFs in genotype comparisons. Subsequently, the WGS-based genotype of the second sample in the sorted list was used as the truth VCF file, and the query VCFs were updated to the remaining batches in the same way until the last sample, resulting in the exhaustive permutation. It is worth noting that there were ${C}_{94}^{2}$ (= 4371) and ${C}_{240}^{2}$ (= 28,680) genotype comparisons after this thorough permutation in the first and second batches, respectively.

It is important to mention that for any mismatched pair of samples, the genotype consistency rate, the number of genetic variants, and the number of true positives (TPs) would remain unaffected irrespective of which sample provided the query or truth data. It is essential to state that the genotype comparison was not based on every possible combination of samples (i.e., ${A}_{94}^{2}$ or ${A}_{240}^{2}$).

### Statistical analysis

To evaluate the difference in genotype consistency rate, the number of genetic variants in truth and query VCF files, and the number of TP in genotype comparisons between matched and mismatched data, one-sided t-tests were applied. It was assumed that these data were higher in matched pairs compared with mismatched pairs. Two-sided t-tests were applied to test the differences in genotype consistency rate between matched pairs of WGS and WGBS VCF files between Fingerprint Panels 6 and 7. All of the t-tests were conducted using R 4.2.2. The distribution of these data was plotted using the R package “vioplot” under default parameter settings.

## Supplementary Information

**Additional file 1.**

**Additional file 2.**

## Data Availability

The WGS data have been deposited in the Genome Sequence Archive for Human (https://ngdc.cncb.ac.cn/gsa/) at the National Genomics Data Center, Beijing Institute of Genomics, Chinese Academy of Sciences, under the accession number (HRA001351). Code, WGBS data, and summary data of this study are available from the corresponding author upon reasonable request.
