# Supplementary material for: A pipeline for sample tagging of whole genome bisulfite sequencing data using genotypes of whole genome sequencing
Source: BMC Genomics. 2023 Jun 23;24:347. doi: 10.1186/s12864-023-09413-2 (PMC10288677; doi:10.1186/s12864-023-09413-2)
Supplement: Supplementary file 2 — Additional file 2. [file 12864_2023_9413_MOESM2_ESM.docx]

**Additional files**


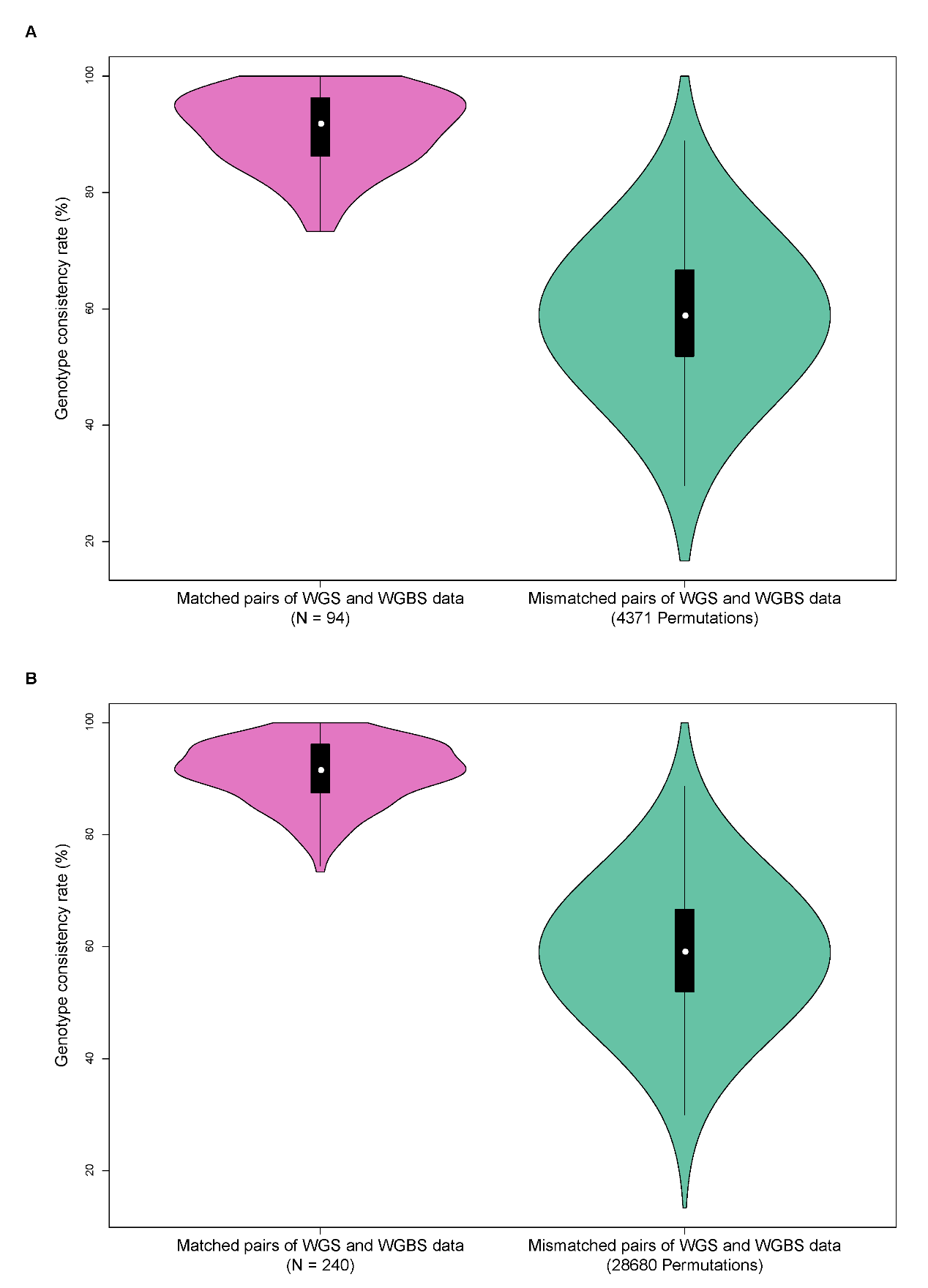


**Supplementary Fig. 1** Violin plots for genotype consistency rate between truth (WGS-based or mass-spectrometry-based) and query (WGBS-based) VCF files using Fingerprint Panel 1. A, genotype consistency rate among the 94 samples in the first batch. Because of the high consistency of genotypes between WGS and mass spectrometry genotyping data for the 52 fingerprint SNPs, genotypes obtained from mass spectrometry were applied as truth data in this comparison of 94 samples. B, genotype consistency rate among the 240 samples in the second batch. Genotypes of the 52 fingerprint SNPs were extracted from WGS data and applied as truth data for this comparison in 240 samples. Genotype consistency rate of matched pairs of WGS and WGBS data was shown in pink, while genotype consistency rate of mismatched pairs of WGS and WGBS data (exhaustive permutation) was shown in light green. The white dot showed median, and the black box showed the interquartile range. 95% confidence interval was shown by the black lines.


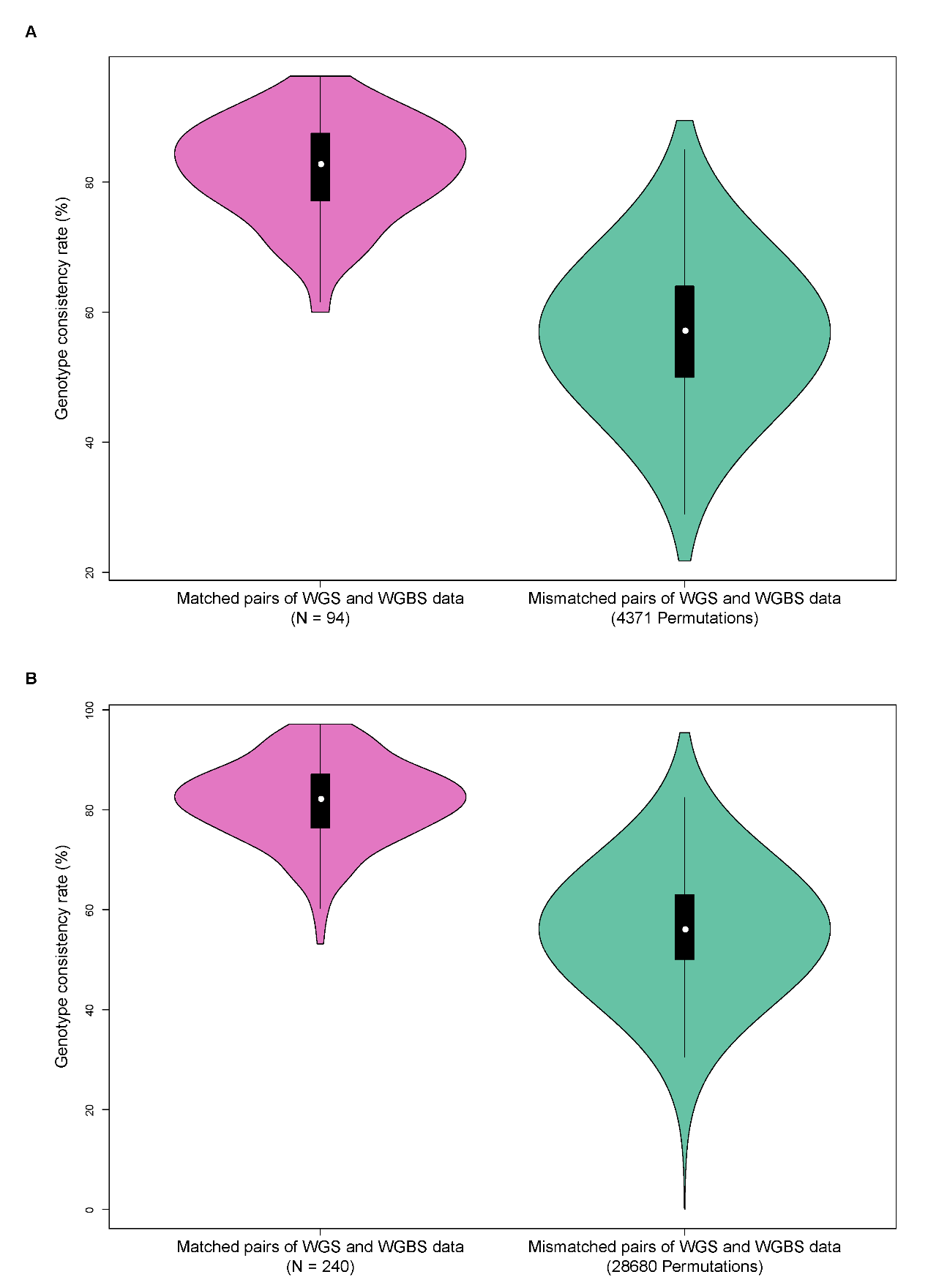


**Supplementary Fig. 2** Violin plots for genotype consistency rate between truth (WGS-based) and query (WGBS-based) VCF files using Fingerprint Panel 2. A, genotype consistency rate among the 94 samples in the first batch. B, genotype consistency rate among the 240 samples in the second batch. Genotype consistency rate of matched pairs of WGS and WGBS data was shown in pink, while genotype consistency rate of mismatched pairs of WGS and WGBS data (exhaustive permutation) was shown in light green. The white dot showed median, and the black box showed the interquartile range. 95% confidence interval was shown by the black lines.


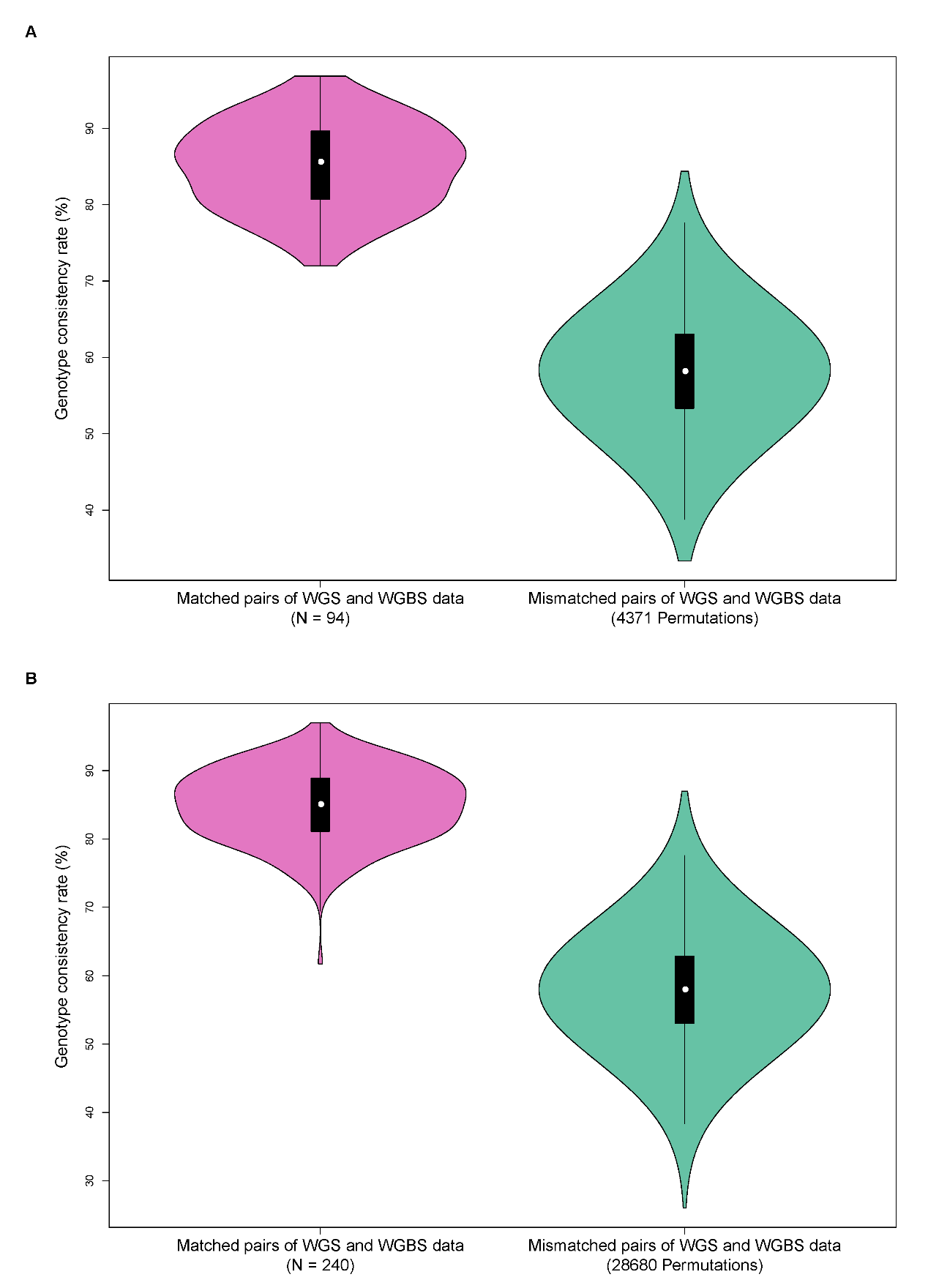


**Supplementary Fig. 3** Violin plots for genotype consistency rate between truth (WGS-based) and query (WGBS-based) VCF files using Fingerprint Panel 3. A, genotype consistency rate among the 94 samples in the first batch. B, genotype consistency rate among the 240 samples in the second batch. Genotype consistency rate of matched pairs of WGS and WGBS data was shown in pink, while genotype consistency rate of mismatched pairs of WGS and WGBS data (exhaustive permutation) was shown in light green. The white dot showed median, and the black box showed the interquartile range. 95% confidence interval was shown by the black lines.


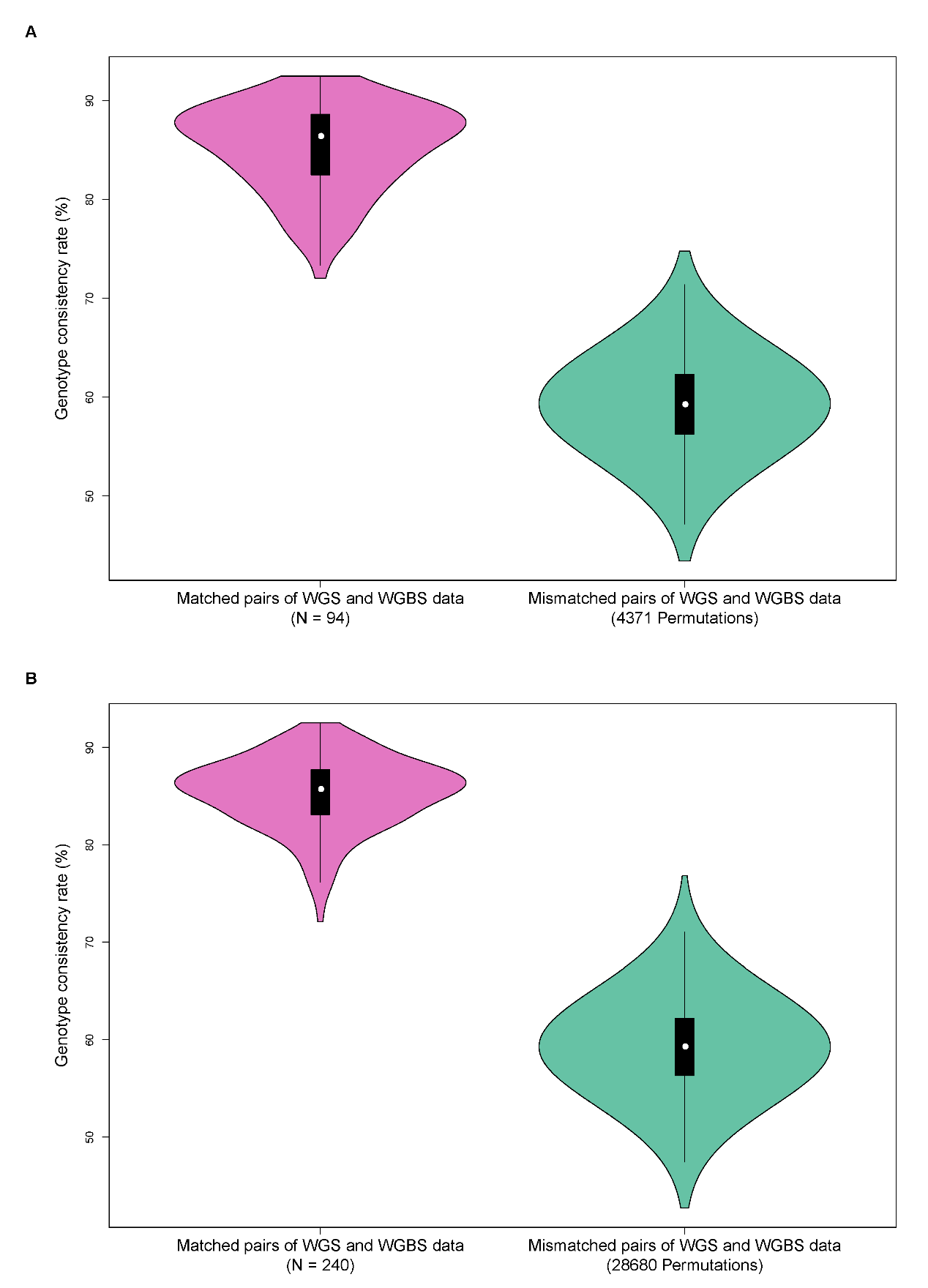


**Supplementary Fig. 4** Violin plots for genotype consistency rate between truth (WGS-based) and query (WGBS-based) VCF files using Fingerprint Panel 4. A, genotype consistency rate among the 94 samples in the first batch. B, genotype consistency rate among the 240 samples in the second batch. Genotype consistency rate of matched pairs of WGS and WGBS data was shown in pink, while genotype consistency rate of mismatched pairs of WGS and WGBS data (exhaustive permutation) was shown in light green. The white dot showed median, and the black box showed the interquartile range. 95% confidence interval was shown by the black lines.


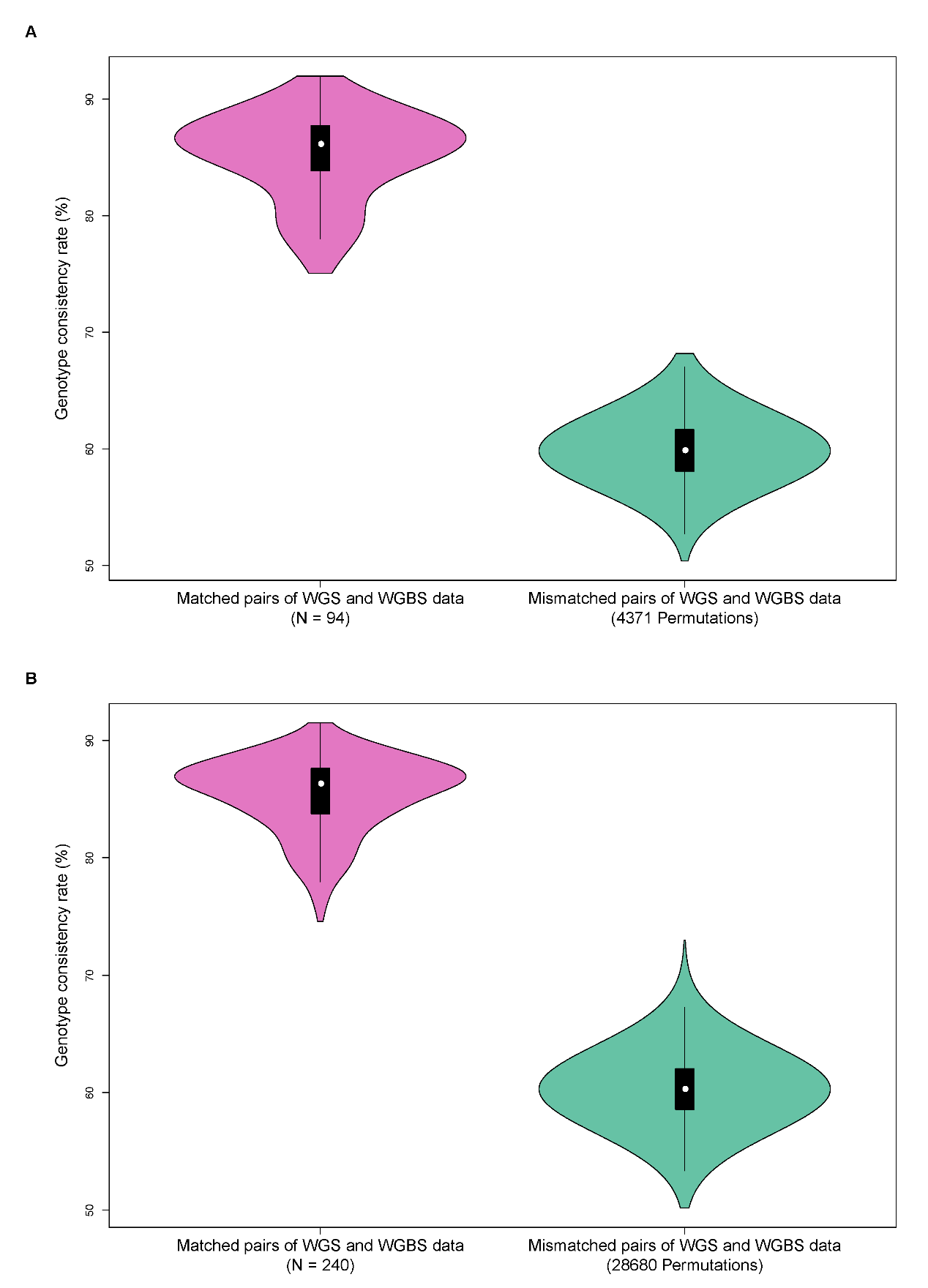


**Supplementary Fig. 5** Violin plots for genotype consistency rate between truth (WGS-based) and query (WGBS-based) VCF files using Fingerprint Panel 5. A, genotype consistency rate among the 94 samples in the first batch. B, genotype consistency rate among the 240 samples in the second batch. Genotype consistency rate of matched pairs of WGS and WGBS data was shown in pink, while genotype consistency rate of mismatched pairs of WGS and WGBS data (exhaustive permutation) was shown in light green. The white dot showed median, and the black box showed the interquartile range. 95% confidence interval was shown by the black lines.


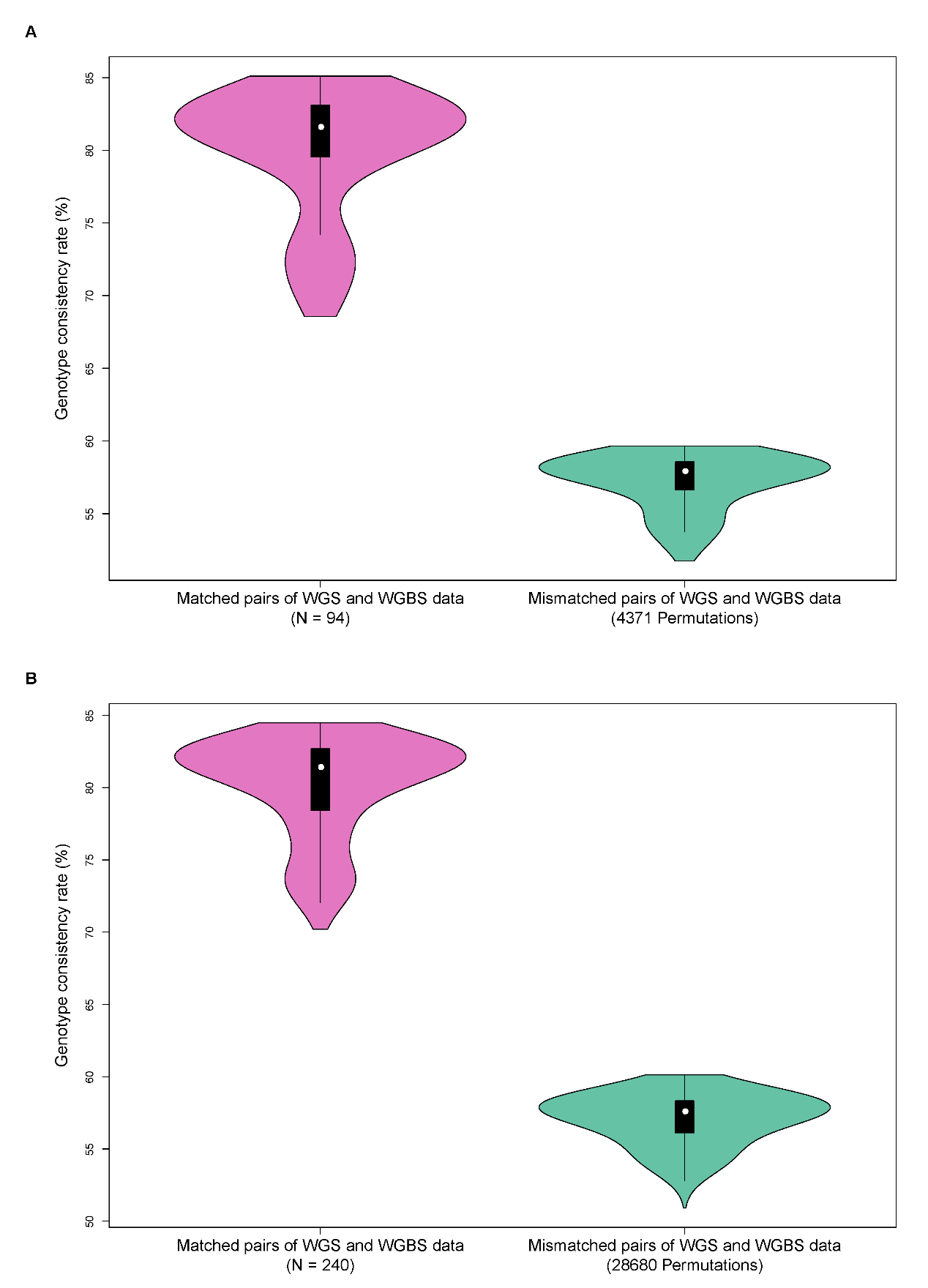
 **Supplementary Fig. 6** Violin plots for genotype consistency rate between truth (WGS-based) and query (WGBS-based) VCF files using Fingerprint Panel 6. A, genotype consistency rate among the 94 samples in the first batch. B, genotype consistency rate among the 240 samples in the second batch. Genotype consistency rate of matched pairs of WGS and WGBS data was shown in pink, while genotype consistency rate of mismatched pairs of WGS and WGBS data (exhaustive permutation) was shown in light green. The white dot showed median, and the black box showed the interquartile range. 95% confidence interval was shown by the black lines.


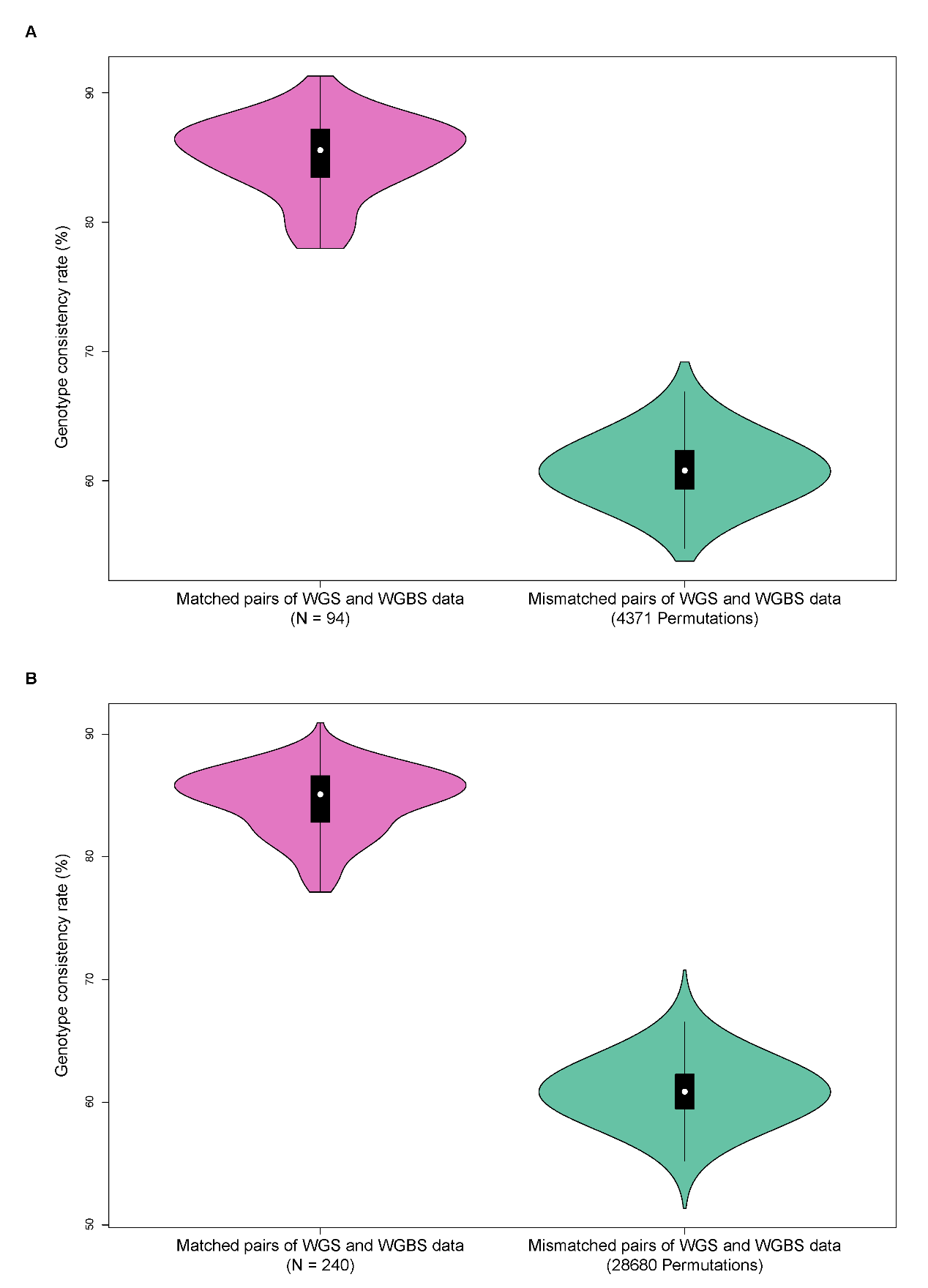


**Supplementary Fig. 7** Violin plots for genotype consistency rate between truth (WGS-based) and query (WGBS-based) VCF files using Fingerprint Panel 8. A, genotype consistency rate among the 94 samples in the first batch. B, genotype consistency rate among the 240 samples in the second batch. Genotype consistency rate of matched pairs of WGS and WGBS data was shown in pink, while genotype consistency rate of mismatched pairs of WGS and WGBS data (exhaustive permutation) was shown in light green. The white dot showed median, and the black box showed the interquartile range. 95% confidence interval was shown by the black lines.


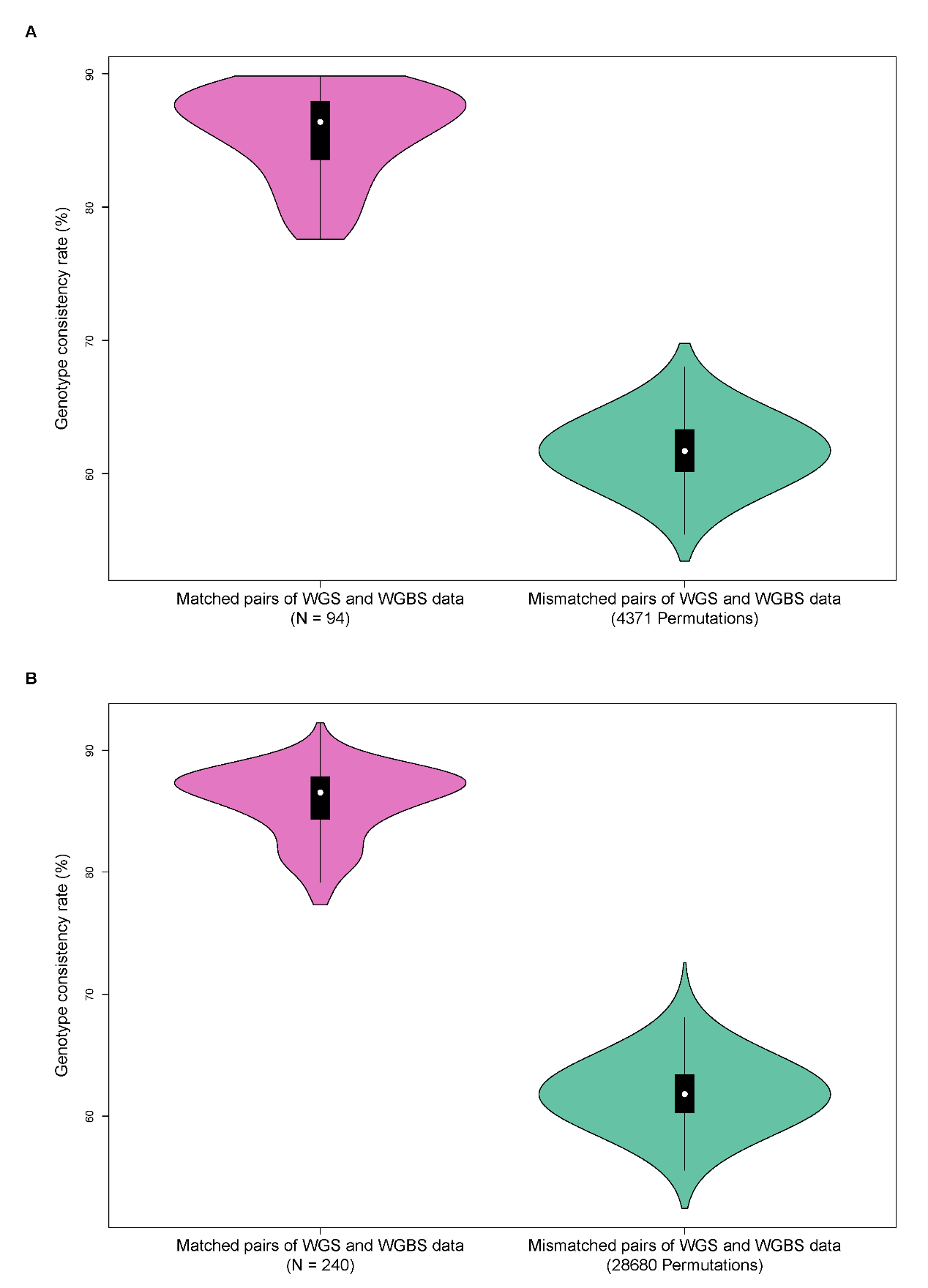


**Supplementary Fig. 8** Violin plots for genotype consistency rate between truth (WGS-based) and query (WGBS-based) VCF files using Fingerprint Panel 9. A, genotype consistency rate among the 94 samples in the first batch. B, genotype consistency rate among the 240 samples in the second batch. Genotype consistency rate of matched pairs of WGS and WGBS data was shown in pink, while genotype consistency rate of mismatched pairs of WGS and WGBS data (exhaustive permutation) was shown in light green. The white dot showed median, and the black box showed the interquartile range. 95% confidence interval was shown by the black lines.


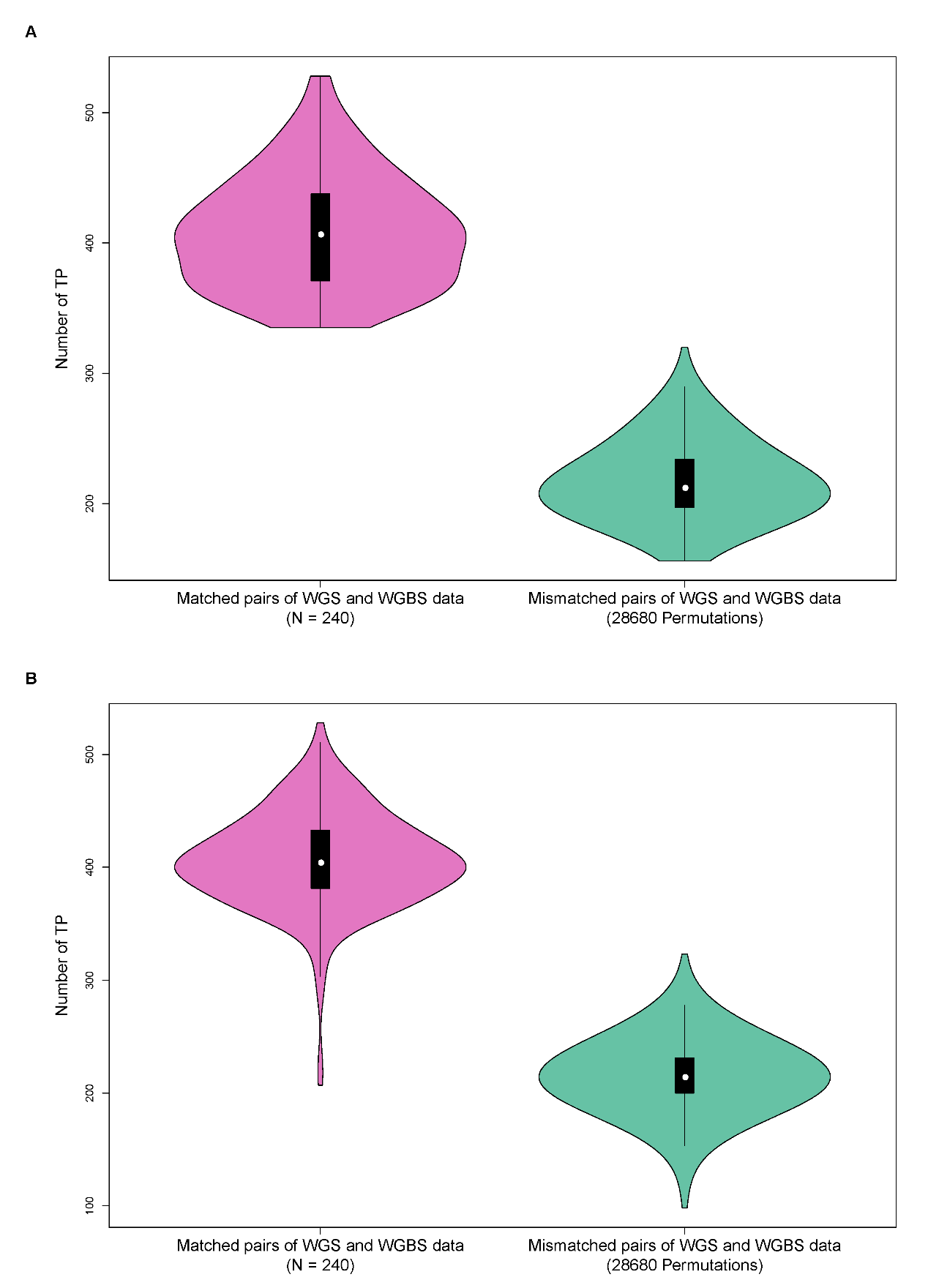


**Supplementary Fig. 9** Violin plots for the number of TP between truth (WGS-based) and query (WGBS-based) VCF files using Fingerprint Panel 5. A, number of TP among the 94 samples in the first batch. B, number of TP among the 240 samples in the second batch. Number of TP for matched pairs of WGS and WGBS data was shown in pink, while number of TP for mismatched pairs of WGS and WGBS data (exhaustive permutation) was shown in light green. The white dot showed median, and the black box showed the interquartile range. 95% confidence interval was shown by the black lines.


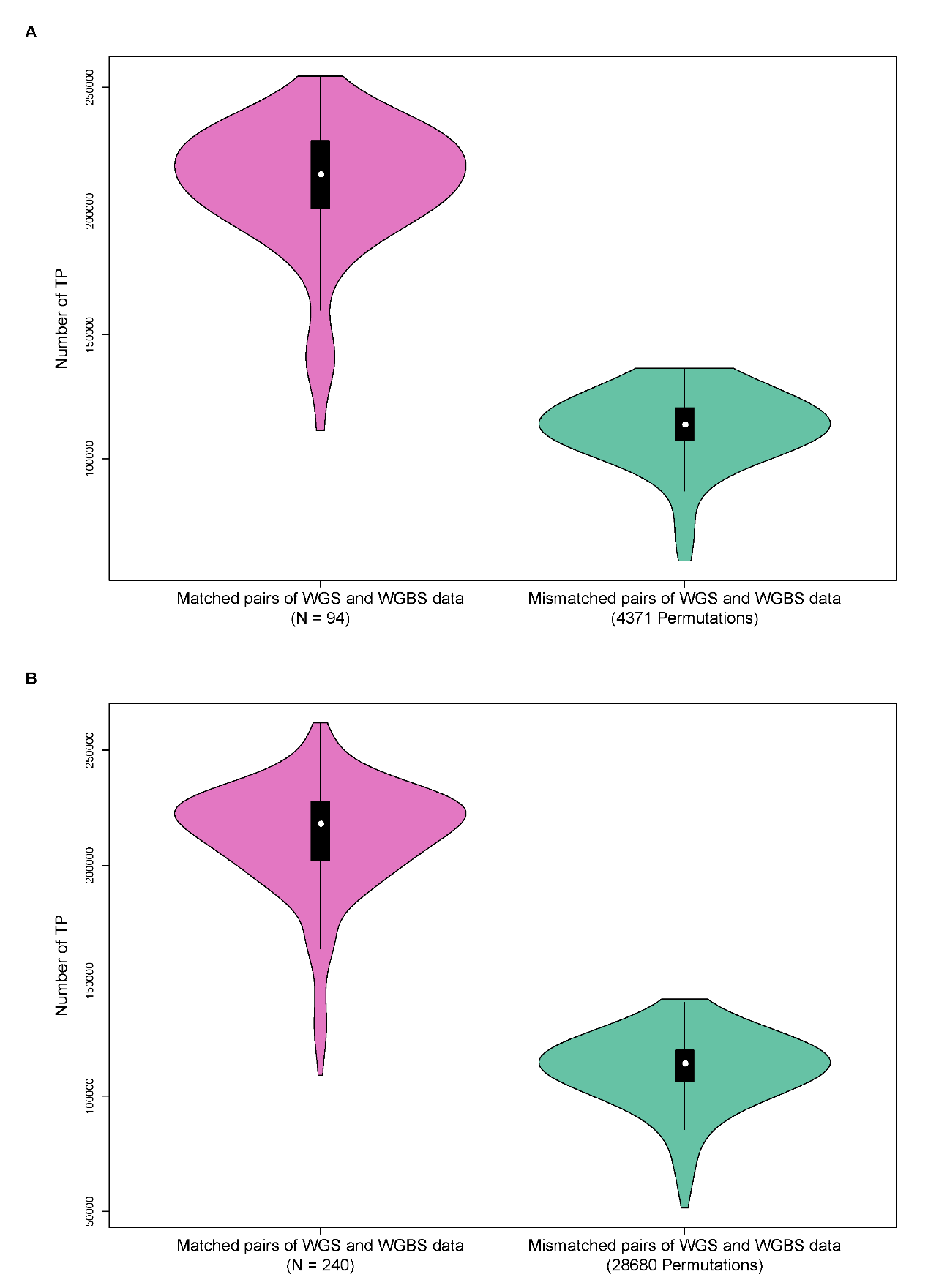


**Supplementary Fig. 10** Violin plots for the number of TP between truth (WGS-based) and query (WGBS-based) VCF files using Fingerprint Panel 6. A, number of TP among the 94 samples in the first batch. B, number of TP among the 240 samples in the second batch. Number of TP for matched pairs of WGS and WGBS data was shown in pink, while number of TP for mismatched pairs of WGS and WGBS data (exhaustive permutation) was shown in light green. The white dot showed median, and the black box showed the interquartile range. 95% confidence interval was shown by the black lines.


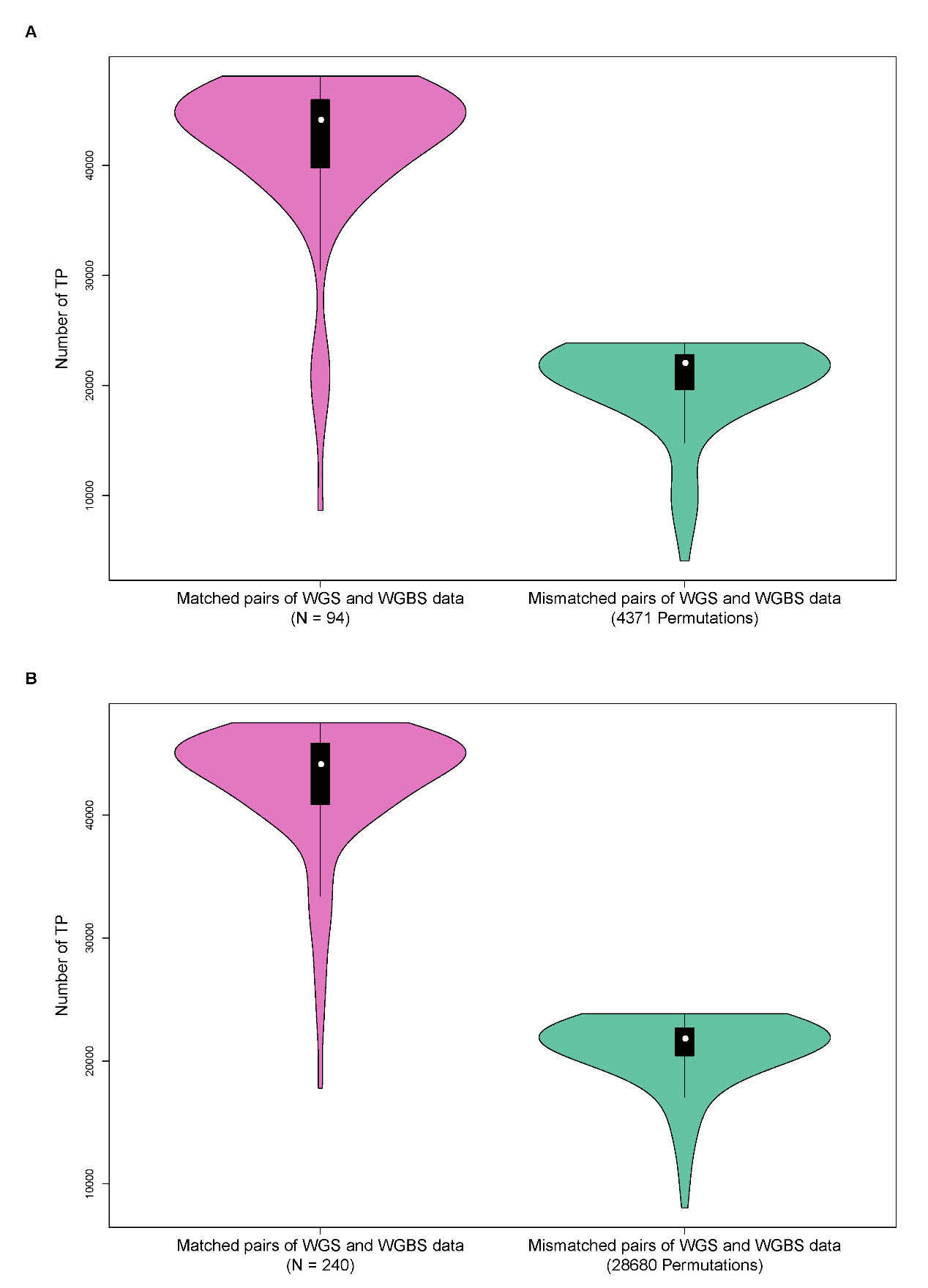


**Supplementary Fig. 11** Violin plots for the number of TP between truth (WGS-based) and query (WGBS-based) VCF files using Fingerprint Panel 7. A, number of TP among the 94 samples in the first batch. B, number of TP among the 240 samples in the second batch. Number of TP for matched pairs of WGS and WGBS data was shown in pink, while number of TP for mismatched pairs of WGS and WGBS data (exhaustive permutation) was shown in light green. The white dot showed median, and the black box showed the interquartile range. 95% confidence interval was shown by the black lines.
